# Supplementary material for: Rapid neurogenesis through transcriptional activation in human stem cells
Source: Mol Syst Biol. 2014 Nov 17;10(11):760. doi: 10.15252/msb.20145508 (PMC4299601; doi:10.15252/msb.20145508)
Supplement: Supplementary file 2 — Supplementary Information [file msb0010-0760-sd2.pdf]

Supplementary Information for "Rapid neurogenesis through transcriptional activation in human stem cells"

Volker Busskamp, Nathan E. Lewis, Patrick Guye, Alex H.M. Ng, Seth L. Shipman, Susan M. Byrne, Neville E. Sanjana, Jernej Murn, Yinqing Li, Shangzhong Li, Michael Stadler, Ron Weiss and George M. Church

Correspondence to George M. Church: gchurch@genetics.med.harvard.edu

Table of contents

- **Supplementary Text**
- **Supplementary Materials and Methods**
- **Supplementary Video S1** (as a separate file)
- **Supplementary References**
- **Supplementary Figures and Legends:** S1 – **S13** (Figures included in Supplementary Information file)
- **Supplementary Tables S1 – S8** (combined in one separate excel file, one spreadsheet per table)

Supplementary Text

Neuronal content cannot explain higher similarity to specific brain regions

To test if some brain regions show higher correlation to our iNGN cells solely for having a higher proportion of neurons in the tissue, we looked deeper into the comparison of brain regions to the 500 most highly differentially expressed gene in our iNGN cells, as reported in the main text. To test this idea, we took GFAP expression levels (a proxy for

glial cell abundance in each tissue sample) and looked to see if the expression is anti-correlated with the Z-scores for each brain region at each time point. If brain region association was the result of higher neuron populations, then one would expect that regions with a higher Z-score would have lower glial cell count (or lower GFAP expression in our analysis). However, only two time points showed anti-correlation: 9 post conception weeks and 37 years, but the p-values were not significant ($p = 0.45$ and $p = 0.2$, respective). This suggests that higher Z-scores in our comparison to the various brain regions analysis do not result from the brain regions having higher neuron populations.

Some details on transcription factors that could help drive the rapid neurogenesis

The rapid acquisition of homogeneous bipolar neurons following Neurogenin induction suggests that there exists a robust gene regulatory network underlying the response. To find transcription factors that contribute to this regulatory network, we analyzed the time-course of mRNA and miRNA expression data in the context of known transcriptional regulatory network processes using Ingenuity's IPA database.

Over the course of differentiation, iNGN cells lost their initial pluripotency. By day 1, common stem cell markers were repressed, as shown in Figure 2A in the main text. By day 1, POU5F1 (OCT4) regulatory targets were differentially expressed ($p = 7.8 \times 10^{-5}$), and changed consistent with decreased POU5F1 activity (Figure 5A). Similarly, expression of targets for NANOG and SOX2 were significantly downregulated ($p < 7.2 \times 10^{-4}$), a finding consistent with the inhibition of their regulatory activities (Figure 5A). Thus, the down regulation of NANOG, POU5F1, and SOX2 is accompanied with the

inhibition of their activities. Our data suggest that by day 1 (Figure 5A), the Neurogenins likely inhibit SOX2, NANOG and POU5F1. In accordance with published work (Evsen et al, 2013; Li et al, 2012; Sun et al, 2001) that suggests that NEUROG2 could inhibit stem cell factors by directly activating NEUROD1 or activating it after binding a p300/CREBBP complex (Sun et al, 2001), we found that downstream targets of these factors were all significantly changing consistent with the possibility of them contributing to the differentiation process. In addition, NEUROD1 activation could also be inhibiting SOX2 (Evsen et al, 2013). Additional transcriptional regulators may reinforce the inhibition of stem cell factors based on our analysis, centered on STAT3 repression. It has been shown that STAT3 phosphorylation maintains pluripotency by enabling the binding to distal enhancers of POU5F1 and NANOG (Do et al, 2013). After day 1, our analysis suggests that STAT3 activity drops considerably in iNGN cells. This might occur as p300/CREBBP is sequestered by NEUROG1 (Sun et al, 2001), which, based on previous literature could indirectly suppress STAT3 activity (Nakashima et al, 1999). This suppression of STAT3 is reinforced as the STAT3 repression of SNAI1 is relieved, leading to the activation of SPARC, which further decreases STAT3 phosphorylation (Bhoopathi et al, 2011). The sequestered p300/CREBBP complex could also fail to activate FOXO1 (Yamagata et al, 2004), which would subsequently be unable to activate POU5F1 and SOX2 (Zhang et al, 2011). In summary, our pathway analysis revealed potential connections of Neurogenins with repression of stem cell factors enabling the destabilization of the stem cell state.

Alongside the increased inhibition of stem cell factors, several neuronal transcription factors are predicted to be activated in our data (Figure 5B). NEUROG1 and NEUROG2

likely activate NEUROD1 (Roybon et al, 2010), a key factor in adult neurogenesis (Gao et al, 2009), and our data suggests that its regulatory functions are strongly activated on day 1 and fortified each day thereafter (Figure 5B). NEUROD1 could subsequently activate other neuronal transcription factors including NEUROD2. The Neurogenin expression also induces neuronal transcription factors, such as ISL1, PAX6, POU3F2, POU4F1, TLX3 and ZEB1. In addition to the activation of many transcription factors, inhibitors of neurogenesis showed suppression of their activity over time, including HES1 and REST. Our transcriptomic data shows increasing inhibition of REST regulation each subsequent day ($p < 0.003$; Figure 5B) that was accompanied by the upregulation of many of its targets. While there are not any known direct connections between the Neurogenins and REST, it is known that REST is activated by POU5F1 and NANOG (Campbell et al, 2007; Soldati et al, 2012) and repressed by NEUROD2 via ZEB1 (Ravanpay et al, 2010). REST suppression would enable activation of a few dozen neuronal genes. Thus, as the Neurogenins activate several core neuronal transcription factors, many downstream neuronal genes were expressed in the iNGN cells resulting in a concerted activation of neuronal fate commitment.

Comparison of singly expressed Neurogenin constructs and the iNGN construct in human iPS cells

In this work, murine Neurog1 and Neurog2 were expressed together from the same construct (iNGN). Previous studies demonstrated that the individual Neurogenins could contribute to neuron differentiation, albeit with lower efficiency (Farah et al, 2000; Stein et al, 2014) or by using a selection marker (Zhang et al, 2013). Here we compared the

differences in gene expression resulting from our iNGN construct and doxycycline-induced constructs with human NEUROG1 or NEUROG2 alone. When this induction was implemented, neurons were obtained from each experimental setup (Figure S13A). While there are considerable similarities in the genes these transcription factors regulate, both had unique genes they regulated (Figure S13 B-D). Indeed, GO term enrichment (Table S9) showed some differences in the classes of genes that were differentially regulated between the two Neurogenins when compared to the construct used in the iNGN cells, suggesting that both transcription factors compliment each other in the iNGN cell line.

Supplementary Materials and Methods

DNA constructs

NEUROG1 was PCR-amplified from a plasmid containing the cDNA (MHS1010-57998, Thermo Scientific, Hudson NH). NEUROG2 was PCR-amplified from a plasmid containing the cDNA (MHS1010-9204603, Thermo Scientific, Hudson NH). The respective PCR products were recombined into pDORN221 using BP clonase, yielding pENTR_L1_hNgn1_L2 and pENTR_L1_hNgn2_L2. Sequencing confirmed the correct DNA sequence.

Microarray analysis

PGP1 iPS cells were transfected with lentiviral particles delivering constructs containing NEUROG1 or NEUROG2. These were induced using doxycycline, and induced neurons differentiated over the course of 4 days in mTeSR media. Subsequently, cells were

harvested, and expression profiles were assayed using Agilent microarrays. Arrays were normalized using quantile normalization and differentially expressed genes were determined using the two sample t-test (FDR < 0.05, with a fold-change greater than a magnitude of $\log_2(1.5)$).

Supplementary Video S1: Representative time-lapse recording of differentiating iNGN cells from day 2 to day 4.

Supplementary References

Bhoopathi P, Chetty C, Dontula R, Gujrati M, Dinh DH, Rao JS, Lakka SS (2011) SPARC stimulates neuronal differentiation of medulloblastoma cells via the Notch1/STAT3 pathway. Cancer research 71: 4908-4919

Campbell PA, Perez-Iratxeta C, Andrade-Navarro MA, Rudnicki MA (2007) Oct4 targets regulatory nodes to modulate stem cell function. PLoS One 2: e553

Do DV, Ueda J, Messerschmidt DM, Lorthongpanich C, Zhou Y, Feng B, Guo G, Lin PJ, Hossain MZ, Zhang W, Moh A, Wu Q, Robson P, Ng HH, Poellinger L, Knowles BB, Solter D, Fu XY (2013) A genetic and developmental pathway from STAT3 to the OCT4-NANOG circuit is essential for maintenance of ICM lineages in vivo. Genes Dev 27: 1378-1390

Evsen L, Sugahara S, Uchikawa M, Kondoh H, Wu DK (2013) Progression of neurogenesis in the inner ear requires inhibition of Sox2 transcription by neurogenin1 and neurod1. J Neurosci 33: 3879-3890

Farah MH, Olson JM, Sucic HB, Hume RI, Tapscott SJ, Turner DL (2000) Generation of neurons by transient expression of neural bHLH proteins in mammalian cells. Development 127: 693-702

Gao Z, Ure K, Ables JL, Lagace DC, Nave KA, Goebbels S, Eisch AJ, Hsieh J (2009) Neurod1 is essential for the survival and maturation of adult-born neurons. Nat Neurosci 12: 1090-1092

Li S, Mattar P, Zinyk D, Singh K, Chaturvedi CP, Kovach C, Dixit R, Kurrasch DM, Ma YC, Chan JA, Wallace V, Dilworth FJ, Brand M, Schuurmans C (2012) GSK3 temporally regulates neurogenin 2 proneural activity in the neocortex. J Neurosci 32: 7791-7805

Nakashima K, Yanagisawa M, Arakawa H, Kimura N, Hisatsune T, Kawabata M, Miyazono K, Taga T (1999) Synergistic signaling in fetal brain by STAT3-Smad1 complex bridged by p300. Science 284: 479-482

Ravanpay AC, Hansen SJ, Olson JM (2010) Transcriptional inhibition of REST by NeuroD2 during neuronal differentiation. Mol Cell Neurosci 44: 178-189

Roybon L, Mastracci TL, Ribeiro D, Sussel L, Brundin P, Li JY (2010) GABAergic differentiation induced by Mash1 is compromised by the bHLH proteins Neurogenin2, NeuroD1, and NeuroD2. Cerebral cortex 20: 1234-1244

Schulz MH, Pandit KV, Lino Cardenas CL, Ambalavanan N, Kaminski N, Bar-Joseph Z (2013) Reconstructing dynamic microRNA-regulated interaction networks. Proc Natl Acad Sci U S A 110: 15686-15691

Soldati C, Bithell A, Johnston C, Wong KY, Teng SW, Beglopoulos V, Stanton LW, Buckley NJ (2012) Repressor element 1 silencing transcription factor couples loss of pluripotency with neural induction and neural differentiation. Stem Cells 30: 425-434

Stein JL, de la Torre-Ubieta L, Tian Y, Parikshak NN, Hernandez IA, Marchetto MC, Baker DK, Lu D, Hinman CR, Lowe JK, Wexler EM, Muotri AR, Gage FH, Kosik KS, Geschwind DH (2014) A quantitative framework to evaluate modeling of cortical development by neural stem cells. Neuron 83: 69-86

Sun Y, Nadal-Vicens M, Misono S, Lin MZ, Zubiaga A, Hua X, Fan G, Greenberg ME (2001) Neurogenin promotes neurogenesis and inhibits glial differentiation by independent mechanisms. Cell 104: 365-376

Yamagata K, Daitoku H, Shimamoto Y, Matsuzaki H, Hirota K, Ishida J, Fukamizu A (2004) Bile acids regulate gluconeogenic gene expression via small heterodimer partner-mediated repression of hepatocyte nuclear factor 4 and Foxo1. J Biol Chem 279: 23158-23165

Zhang X, Yalcin S, Lee DF, Yeh TY, Lee SM, Su J, Mungamuri SK, Rimmele P, Kennedy M, Sellers R, Landthaler M, Tuschl T, Chi NW, Lemischka I, Keller G, Ghaffari S (2011) FOXO1 is an essential regulator of pluripotency in human embryonic stem cells. Nat Cell Biol 13: 1092-1099

Zhang Y, Pak C, Han Y, Ahlenius H, Zhang Z, Chanda S, Marro S, Patzke C, Acuna C, Covy J, Xu W, Yang N, Danko T, Chen L, Wernig M, Sudhof TC (2013) Rapid single-

step induction of functional neurons from human pluripotent stem cells. Neuron 78: 785-798

Supplementary Figures and Legends

Figure S1 - Extended characterization of the iNGN cell line. (**A**) Schematic outline of the lentiviral constructs used to generate iNGN cells. One construct contains the transcription activator rTA3 that when bound to doxycycline, induces expression of the bicistronic Neurogenin cassette, contained in the second construct. (**B**) Experimental outline: the cells were maintained in defined iPS media (mTeSR1) and doxycycline was applied at day 0. The Rho Kinase inhibitor Y27632 was applied for 24h after plating. (**C**) Contrast images illustrating the induction time course, with the doxycycline induced cells in the upper row and uninduced cells in the lower row. (**D**) Representative transmission light microscopic images of iNGN cells with different lengths of doxycycline treatment, demonstrating that neuron morphology appears to be unaffected by the length of doxycycline treatment; all cells imaged on day 4. (**E**) Representative NANOG immunostaining of induced neurons next to an iPS colony. Transmission light (TM), DAPI and NANOG channels are shown. (**F**) Western Blot analyses for Neurog1, Neurog2, MAP2 and vGLUT1 of induced (+doxycycline) or uninduced (-doxycycline) iNGN samples at day 1 and day 4. ACTB served as a loading control. (**G**) The bicistronic Neurog1+2 construct also rapidly differentiated human ES cells (CHB-8 line, left panel) and another human iPS cell line (PGP9) within four days. Merged channels for DAPI (yellow), MAP2 (cyan) and TUBB3 (magenta) are shown. (**H**) Quantification of MAP2 positive Neurog1+2-induced CHB-8 and PGP9 cells on day 4. The number indicates the number of analyzed cells of three independent biological samples. Scale bars, 20 µm.

Figure S1

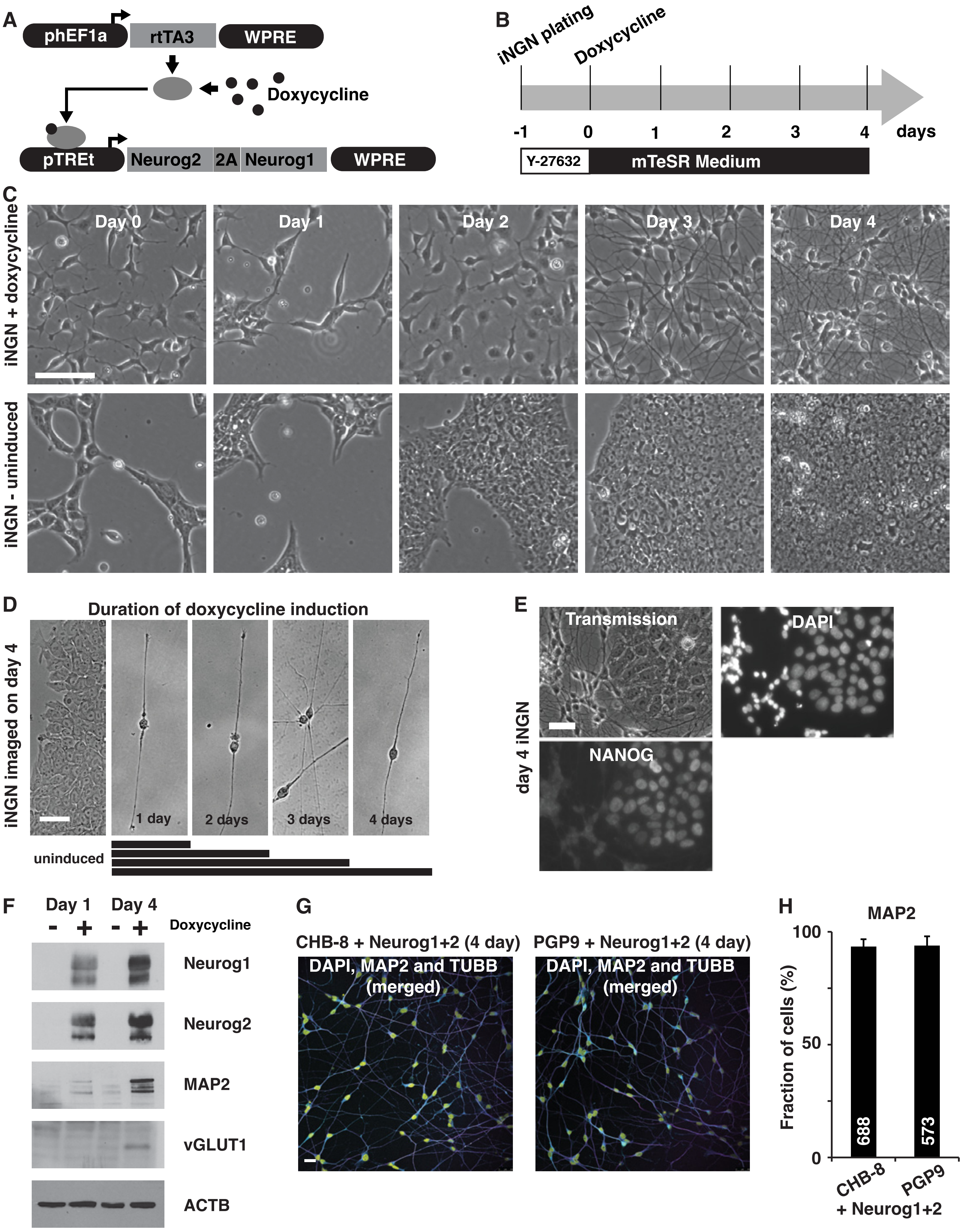
A
phEF1a
rtTA3
WPRE
Doxycycline
pTREt
Neurog2
2A
Neurog1
WPRE
B
iNGN plating
Doxycycline
-1
0
1
2
3
4
days
Y-27632
mTeSR Medium
C
iNGN + doxycycline
iNGN - uninduced
Day 0
Day 1
Day 2
Day 3
Day 4
D
Duration of doxycycline induction
iNGN imaged on day 4
1 day
2 days
3 days
4 days
uninduced
E
Transmission
DAPI
NANOG
day 4 iNGN
F
Day 1
Day 4
Doxycycline
Neurog1
Neurog2
MAP2
vGLUT1
ACTB
G
CHB-8 + Neurog1+2 (4 day)
PGP9 + Neurog1+2 (4 day)
DAPI, MAP2 and TUBB (merged)
H
MAP2
Fraction of cells (%)
100
50
0
688
573
CHB-8
PGP9
+ Neurog1+2

Figure S2 - Neuronal features of iNGN cells. (**A**) Representative immunostainings of iNGN cells after 4 days of induction, for MAP2, DCX, NeuN, GAT3, TUBB3, NCAD, SYN1, SOX2, PAX6, PSD95, GLUR2 and nuclear DAPI dye. The cells are positive for the neuronal markers MAP2, TUBB3, NCAD and NeuN as well as for the neural progenitor markers DCX, SOX2 and PAX6. Synaptic markers such as SYN1, PSD95 and GLUR2 were also detected whereas iNGN cells were immuno-negative for the GABA transporter GAT3. (**B**) Quantification of MAP2, NeuN, GLUR2, PAX6, DCX, SOX2 and GAT3 immuno-positive cells. The number refers to the number of analyzed cells. (**C**) Cell density of induced and uninduced iNGN cells measured by quantifying DAPI signals in biological triplicates at day 4. (**D**) Representative whole-cell voltage recording of an immature iNGN cell cultured entirely in stem cell media on day 4 in response to the injection of current as indicated. (**E**) Representative spontaneous excitatory postsynaptic current recording of a day 42 iNGN cell under neuronal culturing conditions. (**F**) Action potential threshold (in mV), amplitude (in mV), and width (in ms) at 4 days (filled circles) and 14 days (open circles) post-induction. Circles represent individual recordings. Error bars, standard error of the mean (SEM). p-value, $*** \leq 0.001$. Scale bars, 20 µm.

Figure S2

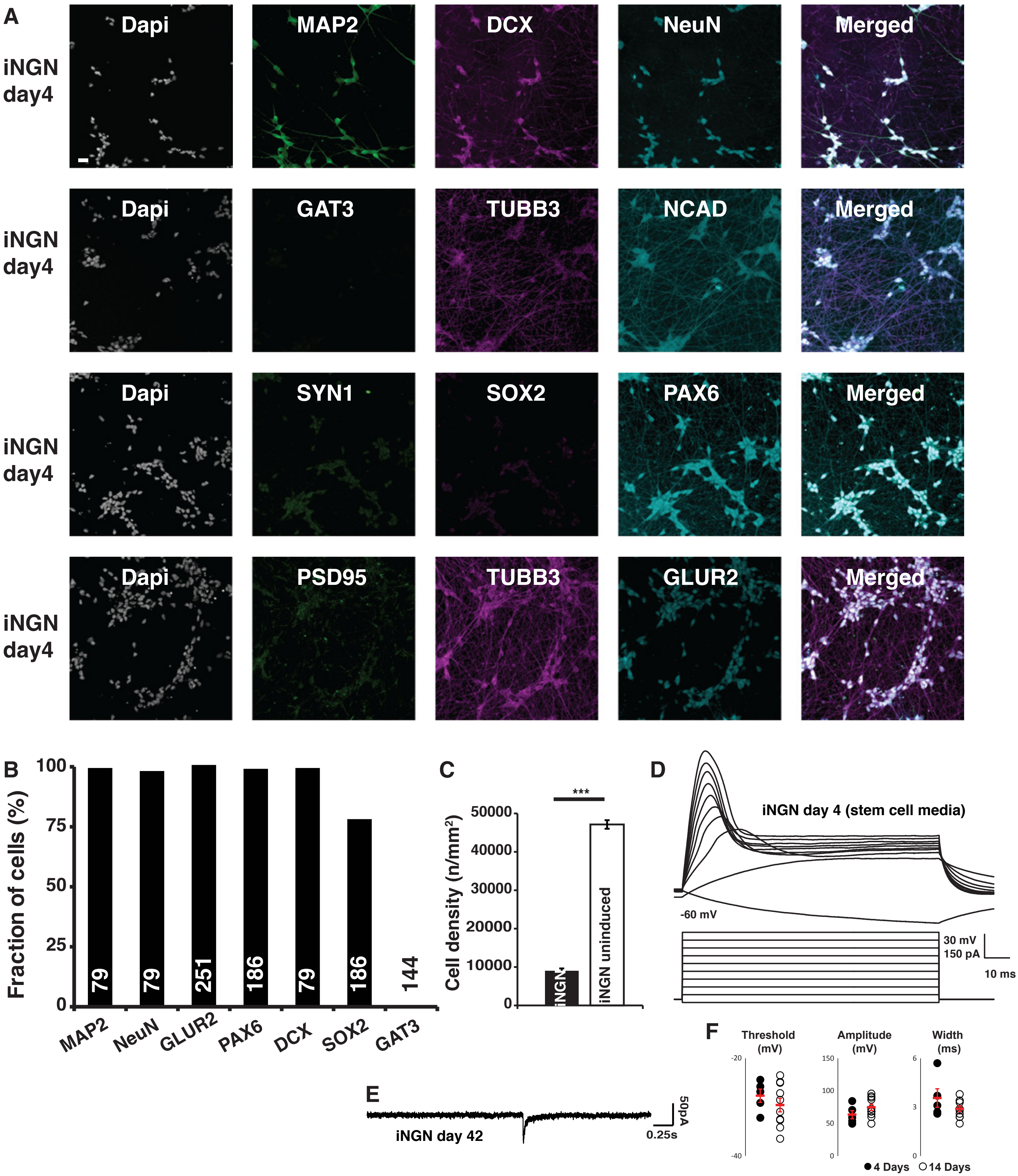
A
iNGN day4
Dapi
MAP2
DCX
NeuN
Merged
GAT3
TUBB3
NCAD
SYN1
SOX2
PAX6
PSD95
GLUR2
B
Fraction of cells (%)
100
75
50
25
0
79
79
251
186
79
186
144
MAP2
NeuN
GLUR2
PAX6
DCX
SOX2
GAT3
C
***
Cell density (n/mm²)
50000
40000
30000
20000
10000
0
iNGN
iNGN uninduced
D
iNGN day 4 (stem cell media)
-60 mV
30 mV
150 pA
10 ms
E
iNGN day 42
50pA
0.25s
F
Threshold (mV)
Amplitude (mV)
Width (ms)
-20
-40
150
100
50
0
6
3
0
4 Days
14 Days

Figure S3 - RNA-Seq statistics. (**A**) Scatter plots comparing biological replicates (R) at each time point. (**B**) Table showing the number of reads per sample.

Figure S3

A

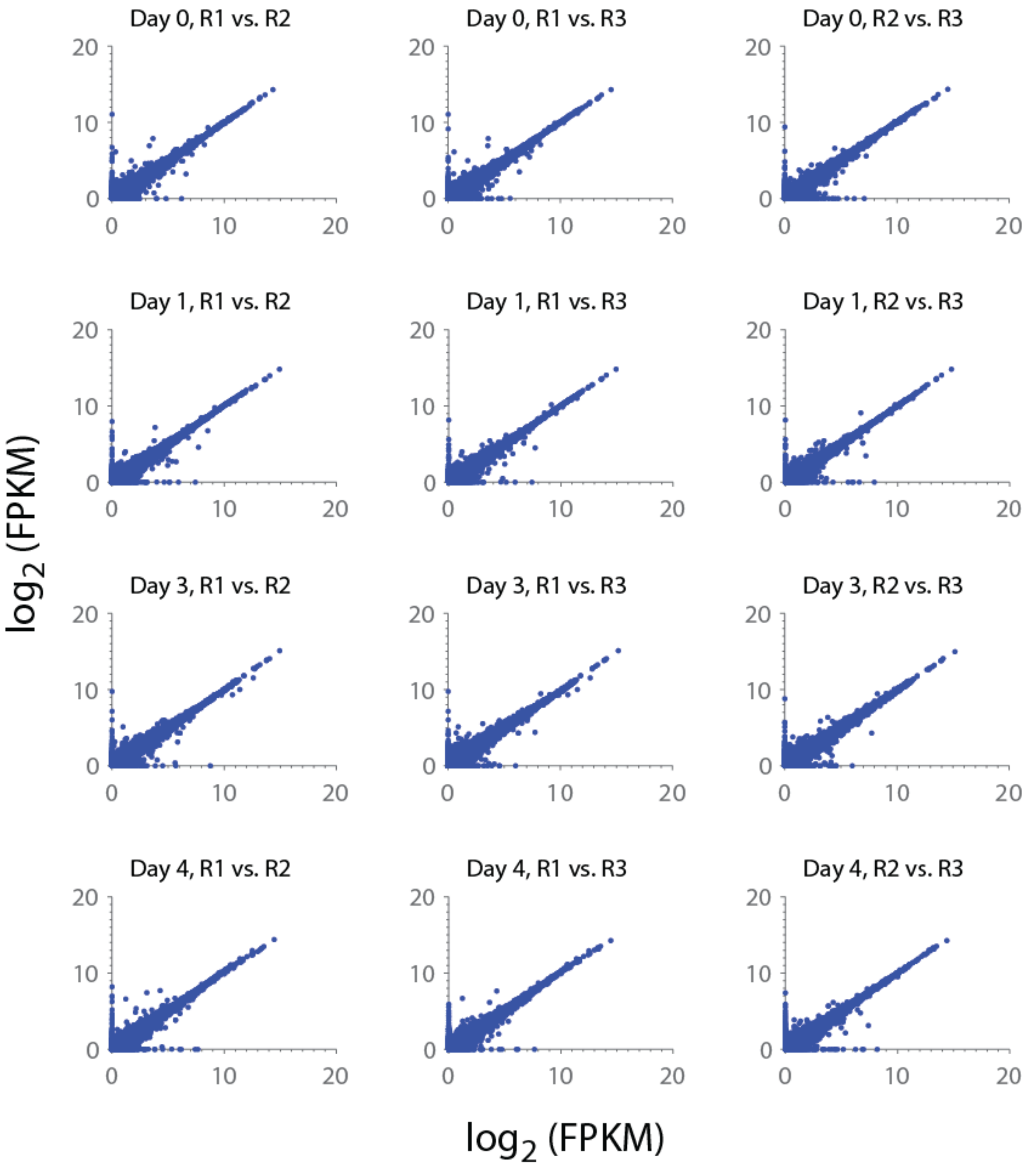

B

Sample:	Total Reads:	Sample:	Total Reads:	Sample:	Total Reads:	Sample:	Total Reads:
Day 0-R1	44,476,956	Day 1-R1	39,152,322	Day 3-R1	34,838,784	Day 4-R1	44,028,220
Day 0-R2	35,754,396	Day 1-R2	47,420,988	Day 3-R2	39,168,634	Day 4-R2	40,587,180
Day 0-R3	40,372,848	Day 1-R3	42,688,716	Day 3-R3	35,508,036	Day 4-R3	39,403,714

Figure S4 - Extended expression analyses of neuronal features. (**A-C**) Expression levels of gene categories over days 1-4. Genes associated with the axon hillock and initial segment are significantly upregulated, including transcripts for cell adhesion and ion channel scaffolding proteins (**A**), voltage-gated sodium channels (**B**), and voltage-gated potassium channels (**C**). Most genes in the Gene Ontology classification of "Synapse assembly" (**D**) are significantly upregulated on day 4 whereas genes of "Cell fate determination" (**E**) pulse in activation between day 1 and 3. Heat maps represent the Z-score for average expression levels for all isoforms over time. (**F**) Gene expression levels of neural progenitor markers measured by RNA-Seq in Fragments Per Kilobase of transcript per Million mapped reads (FPKM). Error bars represent the 95% confidence interval of the abundance of the gene.

Figure S4

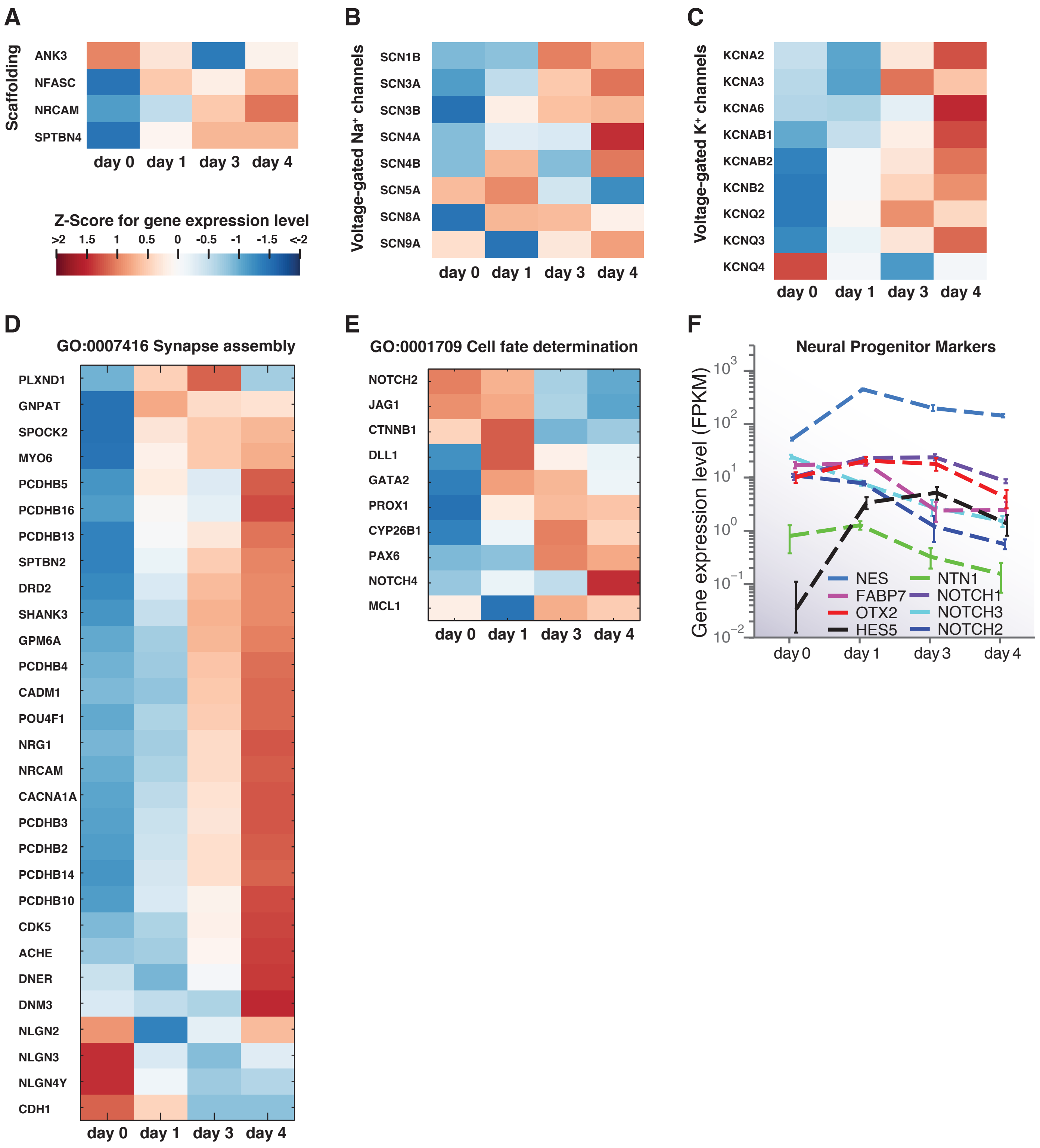

A
Scaffolding
ANK3
NFASC
NRCAM
SPTBN4
day 0 day 1 day 3 day 4
Z-Score for gene expression level
>2 1.5 1 0.5 0 -0.5 -1 -1.5 <-2
B
Voltage-gated Na+ channels
SCN1B
SCN3A
SCN3B
SCN4A
SCN4B
SCN5A
SCN8A
SCN9A
day 0 day 1 day 3 day 4
C
Voltage-gated K+ channels
KCNA2
KCNA3
KCNA6
KCNAB1
KCNAB2
KCNB2
KCNQ2
KCNQ3
KCNQ4
day 0 day 1 day 3 day 4
D
GO:0007416 Synapse assembly
PLXND1
GNPAT
SPOCK2
MYO6
PCDHB5
PCDHB16
PCDHB13
SPTBN2
DRD2
SHANK3
GPM6A
PCDHB4
CADM1
POU4F1
NRG1
NRCAM
CACNA1A
PCDHB3
PCDHB2
PCDHB14
PCDHB10
CDK5
ACHE
DNER
DNM3
NLGN2
NLGN3
NLGN4Y
CDH1
day 0 day 1 day 3 day 4
E
GO:0001709 Cell fate determination
NOTCH2
JAG1
CTNNB1
DLL1
GATA2
PROX1
CYP26B1
PAX6
NOTCH4
MCL1
day 0 day 1 day 3 day 4
F
Neural Progenitor Markers
Gene expression level (FPKM)
10^3
10^2
10^1
10^0
10^-1
10^-2
NES
FABP7
OTX2
HES5
NTN1
NOTCH1
NOTCH3
NOTCH2
day 0 day 1 day 3 day 4

Figure S5 - Quantitative RT-PCR (qRT-PCR) for selected miRNAs in biological triplicates. (**A**) Relative fold-changes of miR-124 over the course of iNGN differentiation. (**B**) miRNA levels normalized to miR-302a expression levels at day 0. (**C-F**) Relative fold-changes of mir-302a (**C**), miR-9 (**D**), miR-96 (**E**) and miR-103 (**F**) over the course of iNGN differentiation. qRT-PCR (black) and nCounter fold-changes (red) are shown; Pearson correlation coefficients r and p-values are indicated, demonstrating good correlations between nCounter and qPCR expression levels. (**G**, **H**) Heatmaps of log_2 miRNA counts assayed by nCounter technology of downregulated (**G**) and upregulated (**H**) miRNAs (miR-124^x refers to expression estimated from qPCR). Only miRNAs that are significantly differentially expressed between day 0 to day 4 and that reach a threshold of at least 500 counts on the nCounter platform at one given time point are shown, q-values < 0.05. qRT-PCR data was normalized to 5S rRNA.

Figure S5

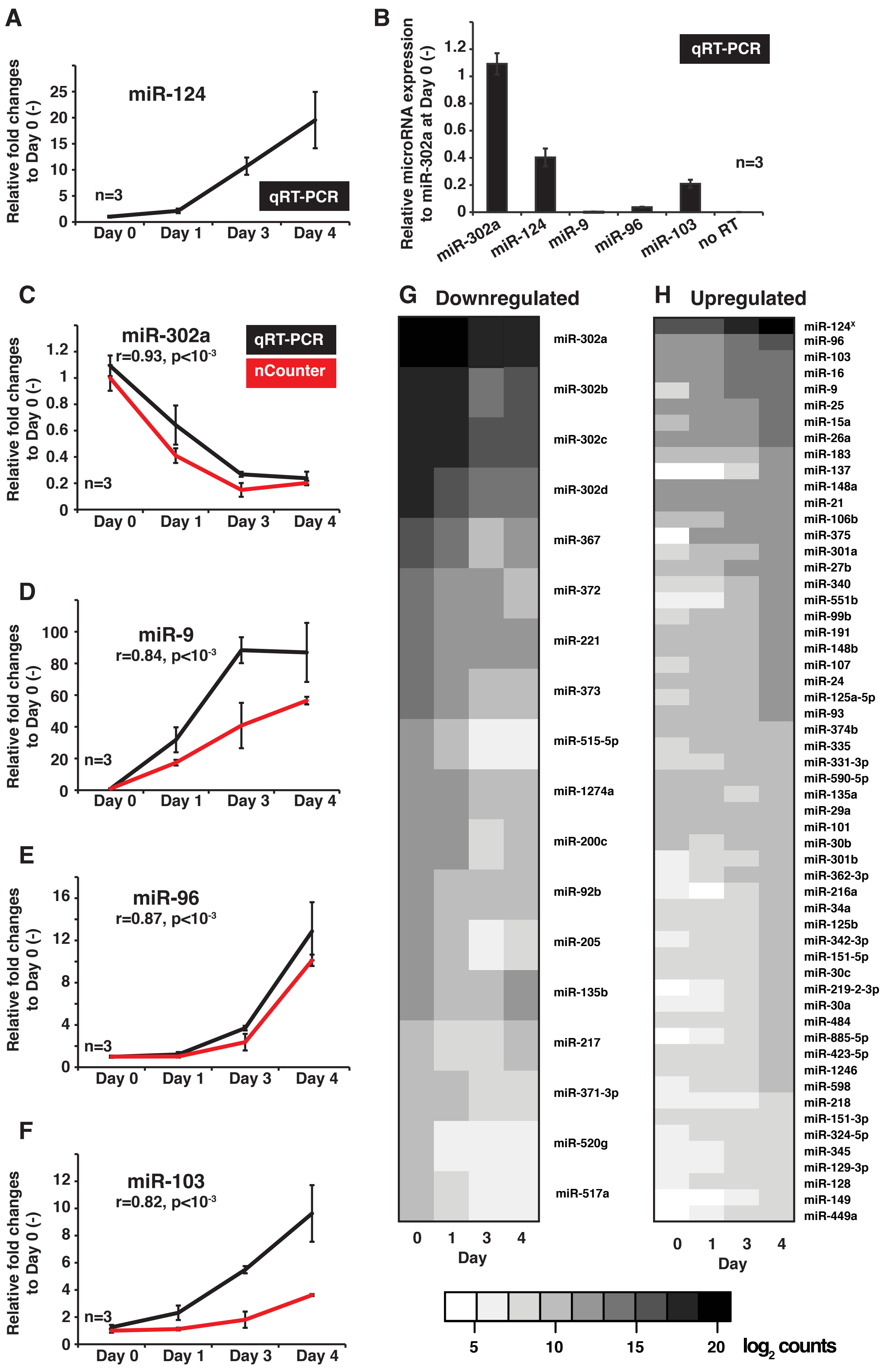
A
Relative fold changes to Day 0 (-)
miR-124
n=3
qRT-PCR
Day 0
Day 1
Day 3
Day 4
B
Relative microRNA expression to miR-302a at Day 0 (-)
qRT-PCR
n=3
miR-302a
miR-124
miR-9
miR-96
miR-103
no RT
C
miR-302a
r=0.93, p<10-3
qRT-PCR
nCounter
D
miR-9
r=0.84, p<10-3
E
miR-96
r=0.87, p<10-3
F
miR-103
r=0.82, p<10-3
G Downregulated
miR-302a
miR-302b
miR-302c
miR-302d
miR-367
miR-372
miR-221
miR-373
miR-515-5p
miR-1274a
miR-200c
miR-92b
miR-205
miR-135b
miR-217
miR-371-3p
miR-520g
miR-517a
H Upregulated
miR-124x
miR-96
miR-103
miR-16
miR-9
miR-25
miR-15a
miR-26a
miR-183
miR-137
miR-148a
miR-21
miR-106b
miR-375
miR-301a
miR-27b
miR-340
miR-551b
miR-99b
miR-191
miR-148b
miR-107
miR-24
miR-125a-5p
miR-93
miR-374b
miR-335
miR-331-3p
miR-590-5p
miR-135a
miR-29a
miR-101
miR-30b
miR-301b
miR-362-3p
miR-216a
miR-34a
miR-125b
miR-342-3p
miR-151-5p
miR-30c
miR-219-2-3p
miR-30a
miR-484
miR-885-5p
miR-423-5p
miR-1246
miR-598
miR-218
miR-151-3p
miR-324-5p
miR-345
miR-129-3p
miR-128
miR-149
miR-449a
Day
log2 counts

Figure S6 - The dynamics of gene expression, analyzed in an effort to identify miRNAs that could contribute to the regulation of gene expression over time, using mirDREM (Schulz et al, 2013). In this method, gene expression profiles with an average FPKM > 0.5 were obtained for all genes that are known to be regulated by a transcriptional regulator with known regulation. Regulated genes included those provided in the mirDREM package and transcriptional regulatory interactions from Ingenuity IPA. These gene expression profiles were clustered such that significant bifurcations (splits in up/down expression) can be found at each day. Then, predicted and known targets (included in mirDREM and obtained from Ingenuity IPA) are used to identify miRNAs that could explain the bifurcation of gene expression changes. For each bifurcation, miRNAs with a p-value for split contribution < 0.1 are shown.

Figure S6

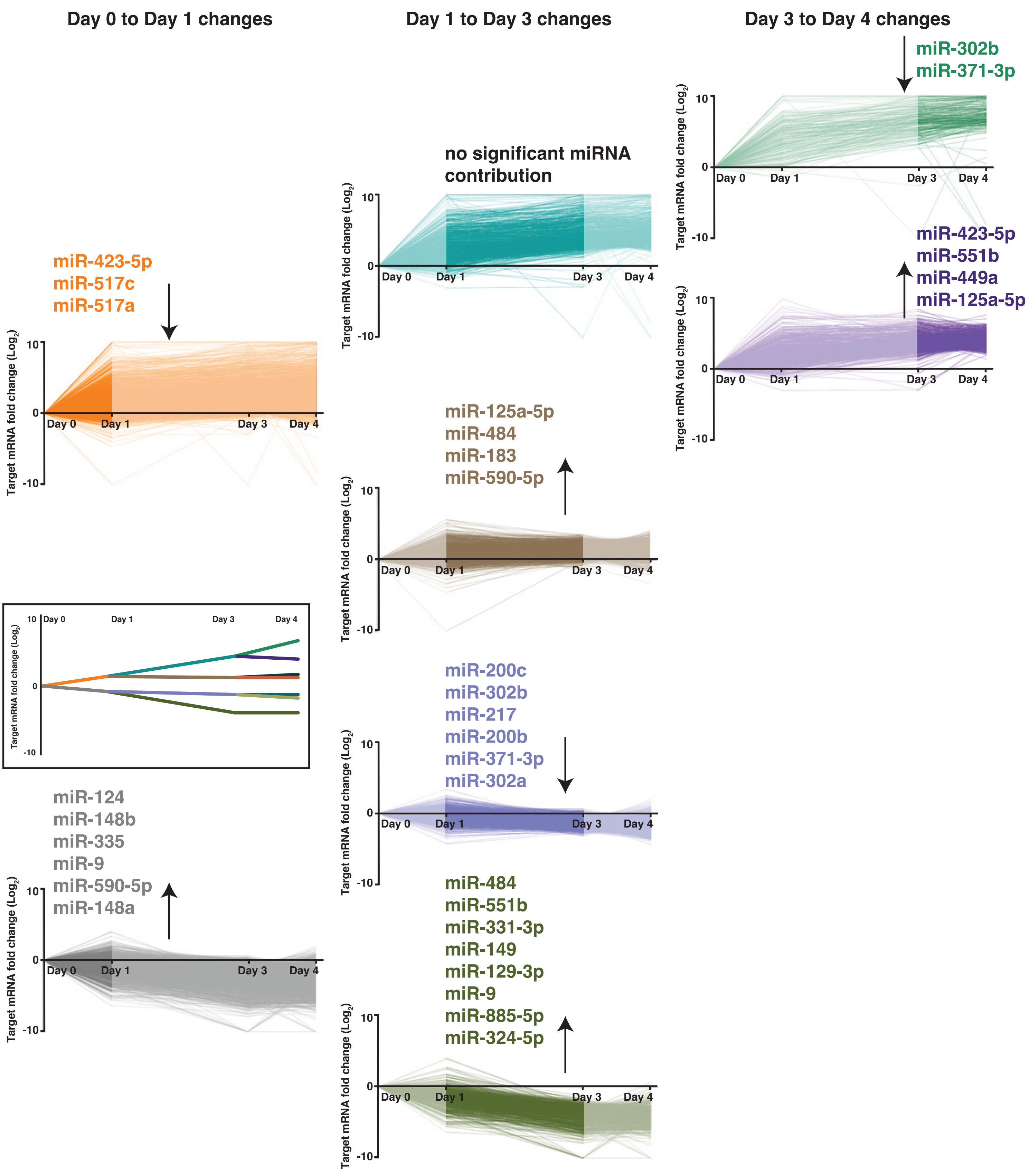

Day 0 to Day 1 changes
Day 1 to Day 3 changes
Day 3 to Day 4 changes
miR-302b
miR-371-3p
no significant miRNA contribution
miR-423-5p
miR-551b
miR-449a
miR-125a-5p
miR-423-5p
miR-517c
miR-517a
miR-125a-5p
miR-484
miR-183
miR-590-5p
miR-200c
miR-302b
miR-217
miR-200b
miR-371-3p
miR-302a
miR-124
miR-148b
miR-335
miR-9
miR-590-5p
miR-148a
miR-484
miR-551b
miR-331-3p
miR-149
miR-129-3p
miR-9
miR-885-5p
miR-324-5p
Target mRNA fold change (Log2)
Day 0
Day 1
Day 3
Day 4
10
0
-10

Figure S7 - Expanded transcription regulatory network during iNGN cell differentiation. Transcription factors were identified that best matched the daily transcriptomic changes. These were used to construct a transcription regulatory network that best explained the mechanisms underlying Neurogenin-mediated neurogenesis (see Materials and methods for details). (**A**) For each day, each transcription factor received an activation Z-score, which describes how consistent the downstream activation or repression was with the experimentally derived knowledge of downstream activation or repression (Ingenuity IPA). For each day, the computed score represents how strongly the downstream genes expression changes support increased activation (positive) or repression (negative) of the upstream transcription factor for that day. (**B**) In addition, transcription factor targets in our regulatory network were associated with miRNAs that were upregulated (red text) or downregulated (blue text). Interactions were based on experimentally validated interactions, as reported in miRTarBase 4.4. The majority of upregulated miRNAs target repressed transcription factors, and most downregulated miRNAs are targeting activated transcription factors. This suggests that the miRNAs are likely contributing to the activity of the regulatory network.

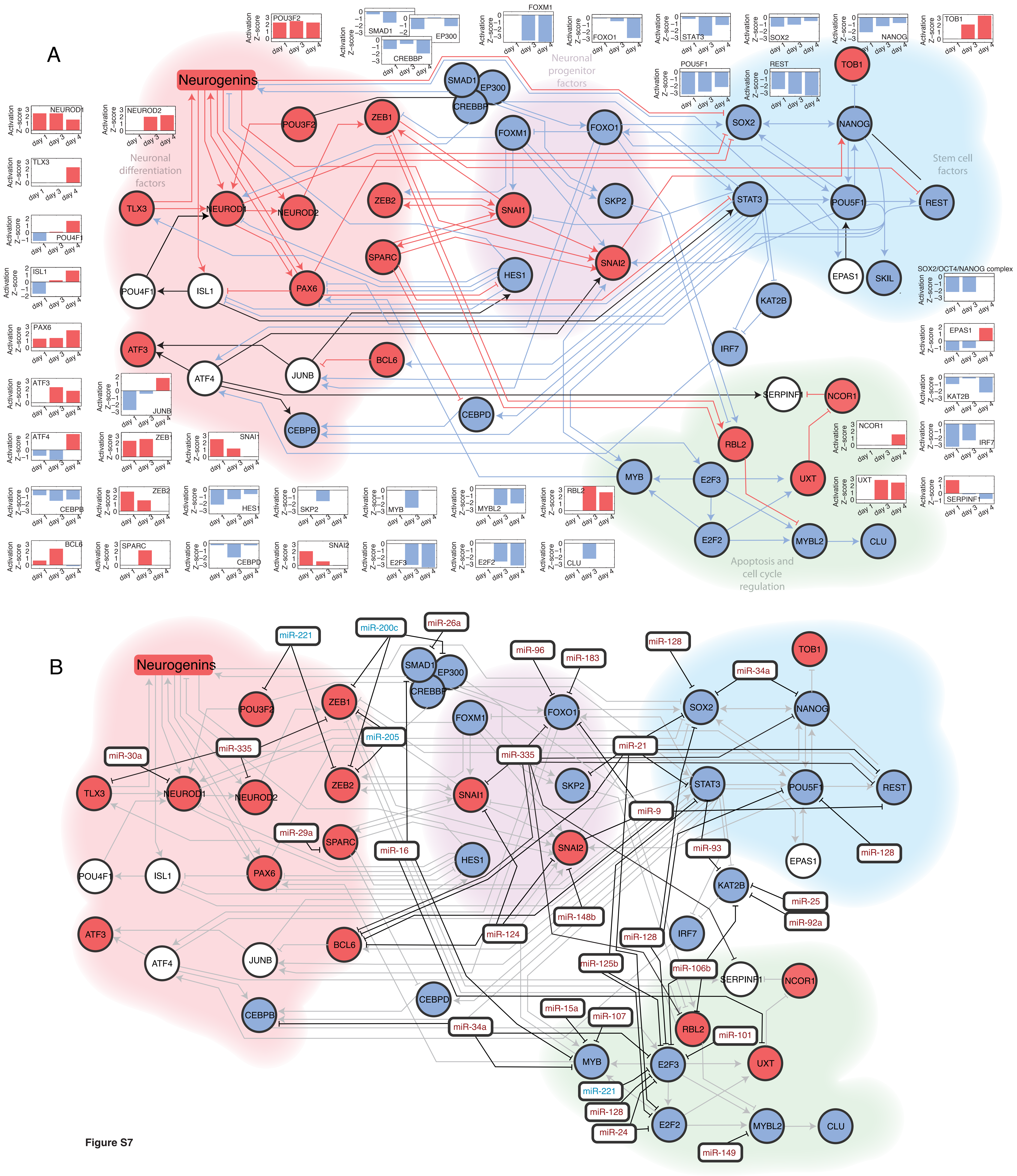
A
B
Neurogenins
Neuronal differentiation factors
Neuronal progenitor factors
Stem cell factors
Apoptosis and cell cycle regulation
SOX2/OCT4/NANOG complex
Activation Z-score
day 1
day 3
day 4
POU3F2
SMAD1
EP300
CREBBP
FOXM1
FOXO1
STAT3
SOX2
NANOG
TOB1
POU5F1
REST
NEUROD1
NEUROD2
TLX3
POU4F1
ISL1
PAX6
ATF3
ATF4
JUNB
CEBPB
CEBPD
BCL6
ZEB1
ZEB2
SNAI1
SNAI2
SPARC
HES1
SKP2
KAT2B
IRF7
EPAS1
SKIL
SERPINF1
NCOR1
RBL2
UXT
MYB
E2F3
E2F2
MYBL2
CLU
miR-221
miR-200c
miR-26a
miR-96
miR-183
miR-128
miR-34a
miR-205
miR-30a
miR-335
miR-21
miR-9
miR-29a
miR-16
miR-93
miR-25
miR-92a
miR-148b
miR-124
miR-125b
miR-106b
miR-15a
miR-107
miR-101
miR-24
miR-149

Figure S7

Figure S8 - Correlation analysis of selected miRNAs and their validated targets. Histograms of correlation coefficients of miR-302abcd (**A**), miR-124 (**B**) miR-96 (**C**), miR-103 (**D**) and miR-9 (**E**) with their validated mRNA targets. miRNAs are repressors of gene expression, therefore one would expect less mRNA expression if a regulating miRNA is upregulated (miR-124, -9, -96 and -103) and increased gene expression if a miRNA is downregulated (miR-302abcd). Negative Pearson correlation coefficients are indicated in blue, positive ones in red. Next to histograms, two examples of each indicated miRNA/validated target pair are shown, miRNA counts (black) and mRNA levels in FPKM (blue) over time.

Figure S8

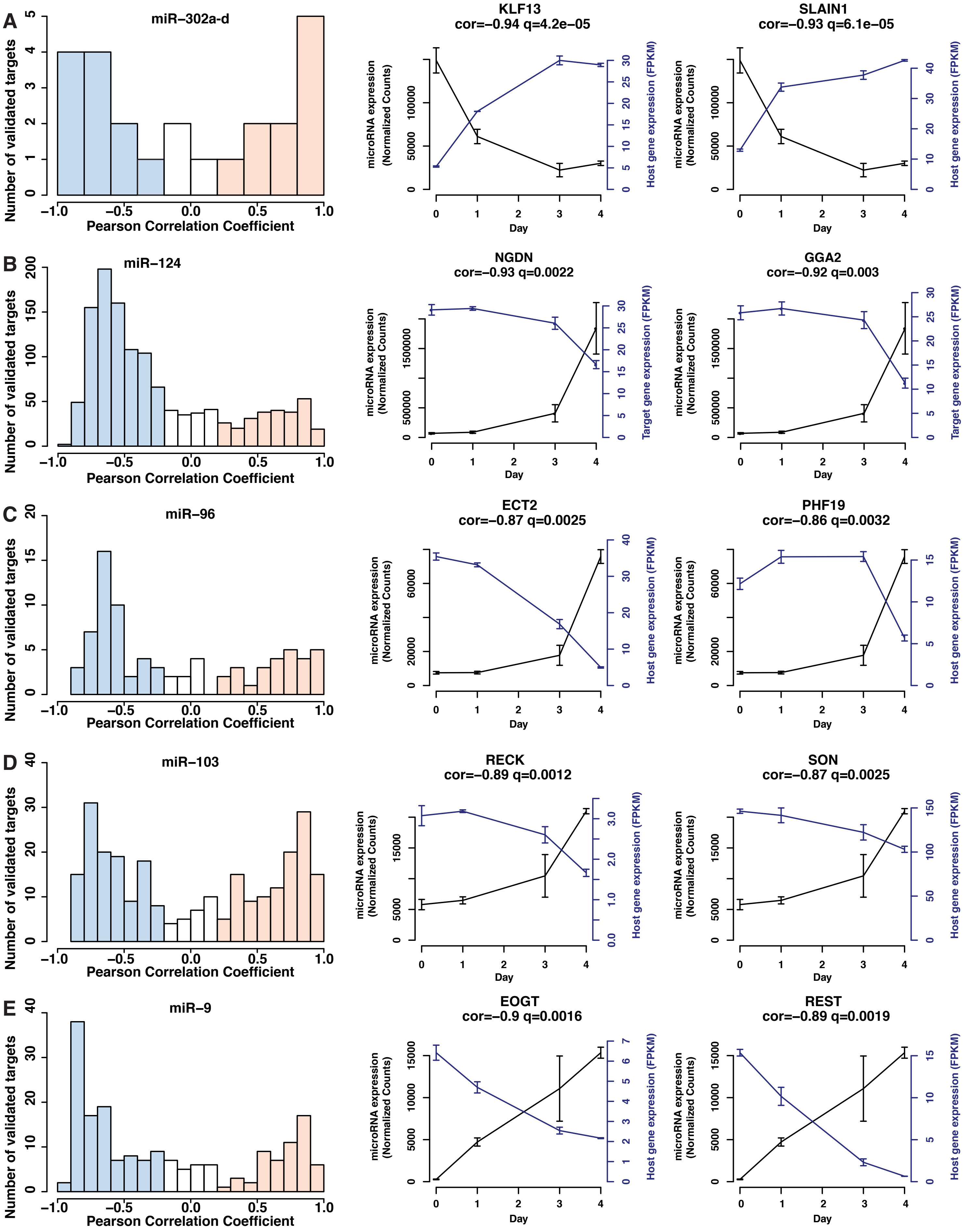

A
miR−302a-d
KLF13
cor=−0.94 q=4.2e−05
SLAIN1
cor=−0.93 q=6.1e−05
B
miR−124
NGDN
cor=−0.93 q=0.0022
GGA2
cor=−0.92 q=0.003
C
miR−96
ECT2
cor=−0.87 q=0.0025
PHF19
cor=−0.86 q=0.0032
D
miR−103
RECK
cor=−0.89 q=0.0012
SON
cor=−0.87 q=0.0025
E
miR−9
EOGT
cor=−0.9 q=0.0016
REST
cor=−0.89 q=0.0019
Number of validated targets
Pearson Correlation Coefficient
microRNA expression (Normalized Counts)
Host gene expression (FPKM)
Target gene expression (FPKM)
Day

Figure S9 - Correlation analysis of miRNA interactions with gene regulatory network members. Pairs of experimentally validated interactions between differentially expressed miRNAs and target transcription factors in our core gene regulatory network were identified, and their expression dynamics were compared. The significance of anti-correlation in expression levels for each miRNA-transcription factor pair was computed. Those showing significant correlation (FDR < 0.05) are plotted in Figure 6, while the remaining pairs are presented here.

Figure S9

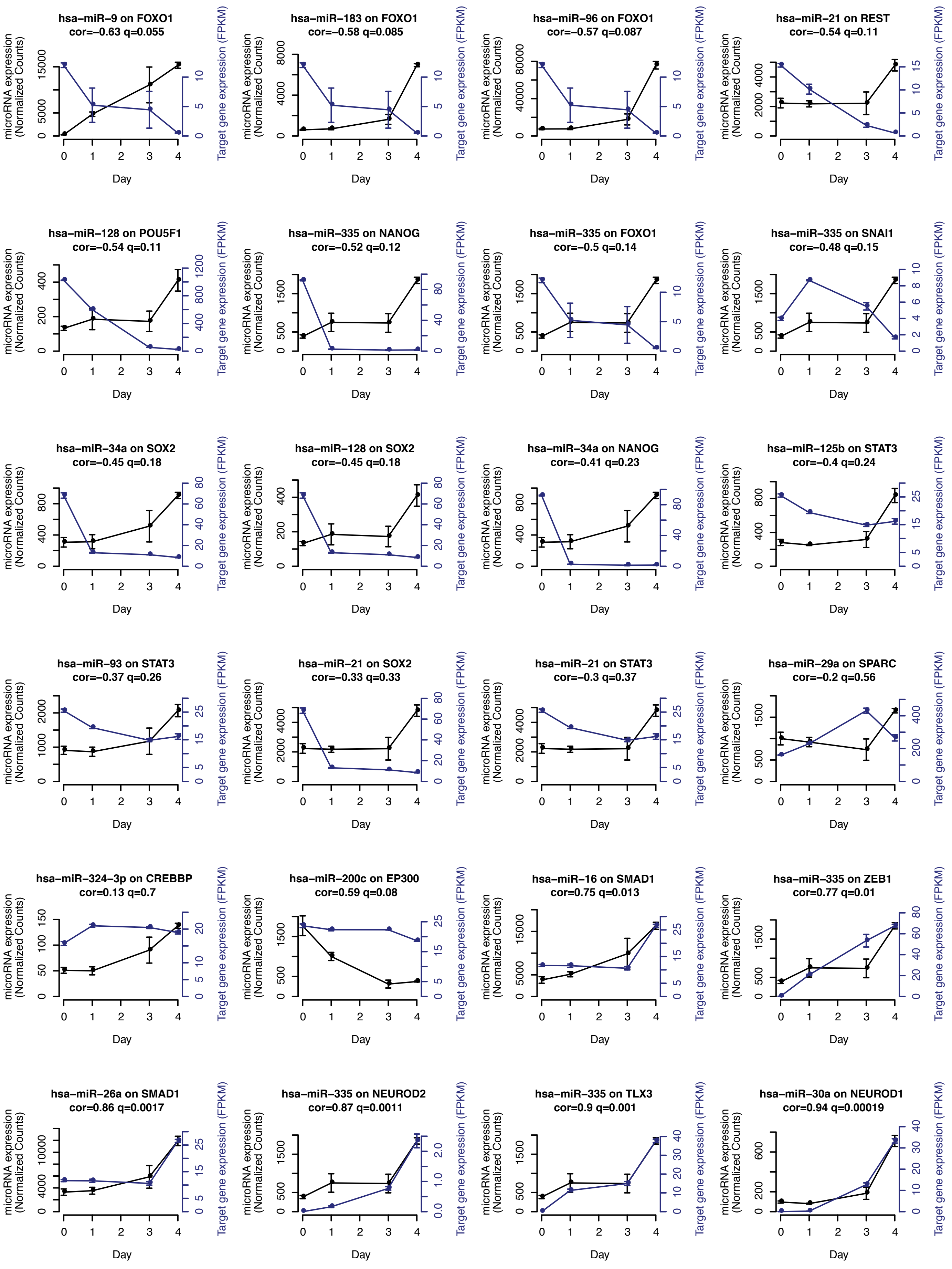
hsa−miR−9 on FOXO1
cor=−0.63 q=0.055
hsa−miR−183 on FOXO1
cor=−0.58 q=0.085
hsa−miR−96 on FOXO1
cor=−0.57 q=0.087
hsa−miR−21 on REST
cor=−0.54 q=0.11
hsa−miR−128 on POU5F1
cor=−0.54 q=0.11
hsa−miR−335 on NANOG
cor=−0.52 q=0.12
hsa−miR−335 on FOXO1
cor=−0.5 q=0.14
hsa−miR−335 on SNAI1
cor=−0.48 q=0.15
hsa−miR−34a on SOX2
cor=−0.45 q=0.18
hsa−miR−128 on SOX2
cor=−0.45 q=0.18
hsa−miR−34a on NANOG
cor=−0.41 q=0.23
hsa−miR−125b on STAT3
cor=−0.4 q=0.24
hsa−miR−93 on STAT3
cor=−0.37 q=0.26
hsa−miR−21 on SOX2
cor=−0.33 q=0.33
hsa−miR−21 on STAT3
cor=−0.3 q=0.37
hsa−miR−29a on SPARC
cor=−0.2 q=0.56
hsa−miR−324−3p on CREBBP
cor=0.13 q=0.7
hsa−miR−200c on EP300
cor=0.59 q=0.08
hsa−miR−16 on SMAD1
cor=0.75 q=0.013
hsa−miR−335 on ZEB1
cor=0.77 q=0.01
hsa−miR−26a on SMAD1
cor=0.86 q=0.0017
hsa−miR−335 on NEUROD2
cor=0.87 q=0.0011
hsa−miR−335 on TLX3
cor=0.9 q=0.001
hsa−miR−30a on NEUROD1
cor=0.94 q=0.00019
microRNA expression (Normalized Counts)
Target gene expression (FPKM)
Day

Figure S10 - Perturbations of miRNA expression by miRNA sponges. (**A-C**) Sponges against the miR-302/367 cluster, miR-124 (**D-F**) and control (**G, H**). The sponge sequences were placed downstream of a GFP-2A-Puromycin cassette that was driven by the EF1α promoter and stably introduced into iNGN cells by lentiviral gene transfer. Untransfected iNGN cells were removed by puromycin selection. (A, D, G) Representative corresponding iNGN cells were stained at day 4 for GFP (green), MAP2 (magenta) and DAPI (white). (**B**, **E**) qPCR analysis over time of KLF13 and TUBB3, two validated miR-302 targets (**B**), as well as NGDN and GGA2, two validated miR-124 targets (**E**), demonstrated increased expression levels in sponged cells (red) versus control samples (black). (**C**, **F**, **H**) Additional representative immunostaings for TUBB3 as well as merged channels for GFP and TUBB3. (**I**) Quantification of non-bipolar control (black) and miRNA-sponged (red) iNGN cells, in biological triplicates. The number of analyzed cells is indicated in the columns. **, p-value ≤ 0.01, *, p-value ≤ 0.05, error bars, SEM, scale bar, 100µm.

Figure S10

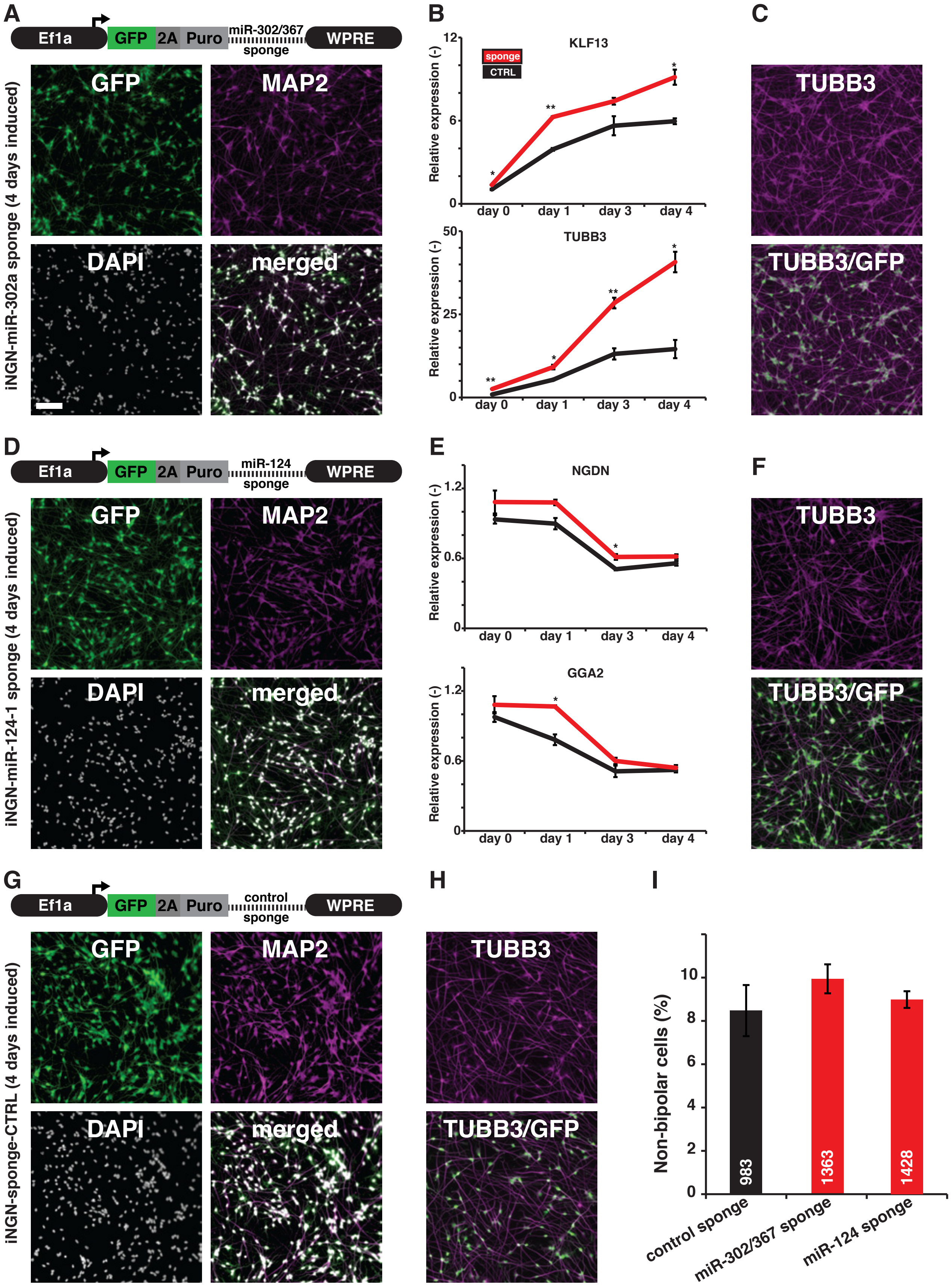
A
Ef1a
GFP
2A
Puro
miR-302/367 sponge
WPRE
iNGN-miR-302a sponge (4 days induced)
GFP
MAP2
DAPI
merged
B
KLF13
sponge
CTRL
Relative expression (-)
12
6
0
day 0
day 1
day 3
day 4
TUBB3
50
25
C
TUBB3
TUBB3/GFP
D
miR-124 sponge
iNGN-miR-124-1 sponge (4 days induced)
E
NGDN
1.2
0.6
GGA2
F
G
control sponge
iNGN-sponge-CTRL (4 days induced)
H
I
Non-bipolar cells (%)
10
8
4
2
983
1363
1428
control sponge
miR-302/367 sponge
miR-124 sponge

Figure S11 - Extended analyses of perturbations of the gene regulatory network. Knockdown experiments using shRNAs (**A**-**C**) and siRNAs (**D**, **E**) against NEUROD1 as well as upregulation of the transcription factor REST (**G**-**I**). qRT-PCR analysis of the NEUROD1 regulated genes NEUROD2 (**A**) and SOX2 (**B**) including a no RT control (**C**). Corresponding gene expression levels over time of NEUROD1-shRNA (red) and sh-CTRL (black) iNGN samples are shown, normalized to ACTB in biological triplicates. (**D**, **E**) Immunohistochemical analysis for TUBB3 and NeuN and nuclear DAPI staining of NEUROD1-siRNA treated iNGN cells (**D**) and control-siRNA treated iNGN cells (**E**) imaged at day 4 of induction. Merged channels are indicated. Scale bar, 50 µm. (**G**) Transmission light images of day 4 REST-overexpressing and control iNGN cells (left) and corresponding uninduced cells (right). The lentiviral doxycycline inducible REST-overexpression construct is shown on the top. (**H**) Quantitative RT-PCR for REST of day 1 induced and uninduced iNGN + REST (black) and iNGN (grey) cells, normalized to ACTB; n refers to the number of biological replicates. (**I**) Day 4 iNGN + REST cells had significantly enlarged soma diameters; n, number of analyzed cells from three independent samples. Scale bar, 20 µm. ***, p-value $\leq$ 0.001. **, p-value $\leq$ 0.01. (**J**) Proportion of neuronal genes (members of neuronal GO terms) under unique NEUROD1 and REST regulation taken from Ingenuity IPA. Error bars, ± SEM.

Figure S11

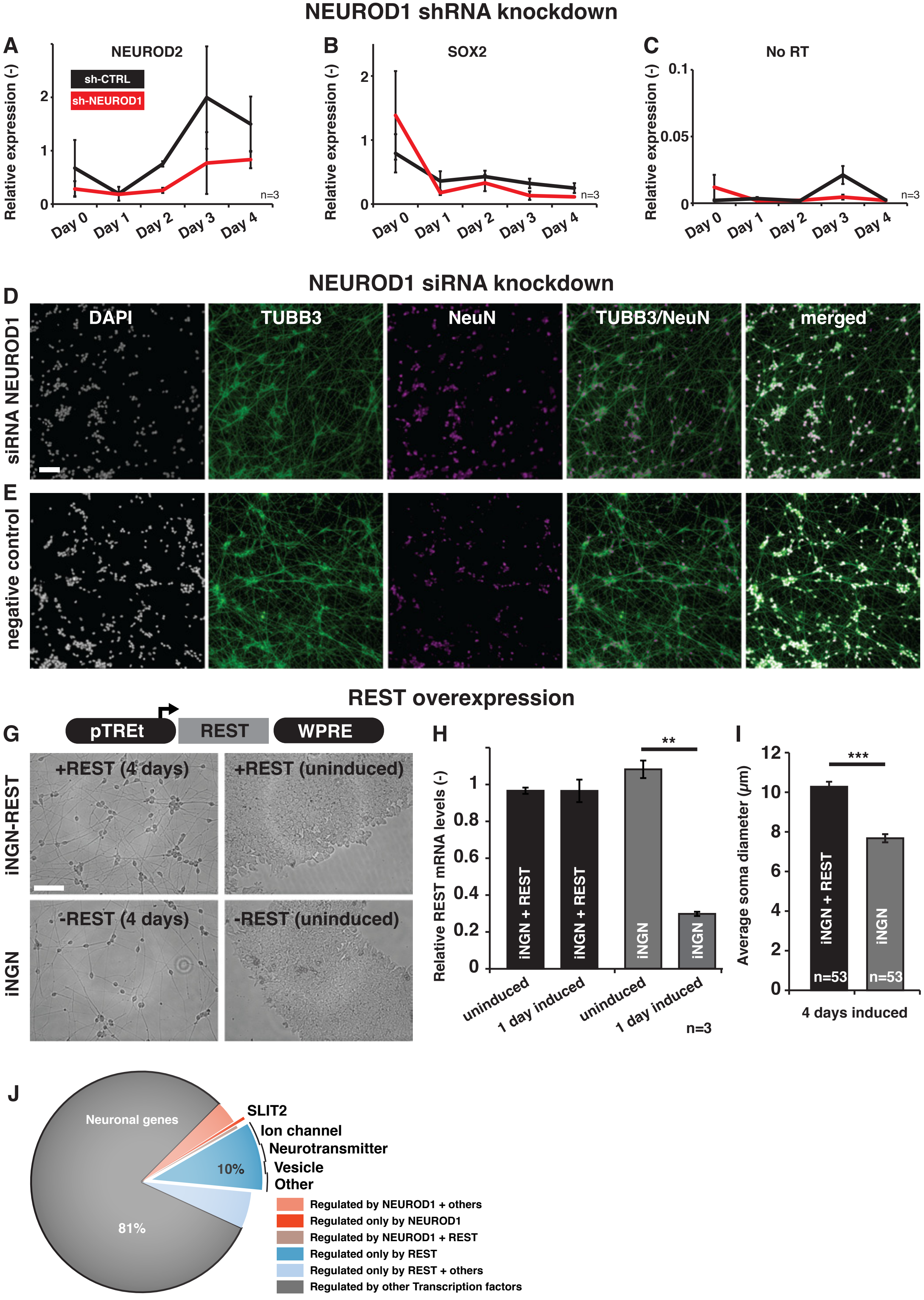

NEUROD1 shRNA knockdown
A
NEUROD2
sh-CTRL
sh-NEUROD1
Relative expression (-)
0
1
2
Day 0
Day 1
Day 2
Day 3
Day 4
n=3
B
SOX2
C
No RT
0.1
0.05
NEUROD1 siRNA knockdown
D
siRNA NEUROD1
DAPI
TUBB3
NeuN
TUBB3/NeuN
merged
E
negative control
REST overexpression
G
pTREt
REST
WPRE
iNGN-REST
iNGN
+REST (4 days)
+REST (uninduced)
-REST (4 days)
-REST (uninduced)
H
Relative REST mRNA levels (-)
0
0.2
0.4
0.6
0.8
1
iNGN + REST
iNGN
**
uninduced
1 day induced
n=3
I
Average soma diameter (μm)
0
2
4
6
8
10
12
***
n=53
4 days induced
J
Neuronal genes
SLIT2
Ion channel
Neurotransmitter
Vesicle
Other
10%
81%
Regulated by NEUROD1 + others
Regulated only by NEUROD1
Regulated by NEUROD1 + REST
Regulated only by REST
Regulated only by REST + others
Regulated by other Transcription factors

Figure S12 - Transient perturbations of core regulatory members. siRNA-knockdown experiments against NEUROD1 (**A**), NEUROD2 (**B**), NEUROD1/NEUROD2 (**C**), NEUROD1/PAX6 (**D**), POU3F2 (**E**) and ZEB1 (**F**) during iNGN differentiation. A scrambled siRNA served as a negative control (NC) shown in (**G**). Uninduced iNGN cells were transfected with siRNAs one day prior doxycycline induction. Contrast images from day 1 to day 4 of induction for each siRNA treatment are shown. Total RNA for qRT-PCR analysis was harvested at day 1 and day 3. The no RT controls served as a measure of DNA contamination. Corresponding gene expression levels at day 1 and day 3 of siRNA knockdown samples (KD) (red, magenta) and NC (black, grey) iNGN samples are shown, normalized to ACTB in biological triplicates. **, p-value ≤ 0.01, *, p-value ≤ 0.05, scale bar, 20µm.

Figure S12

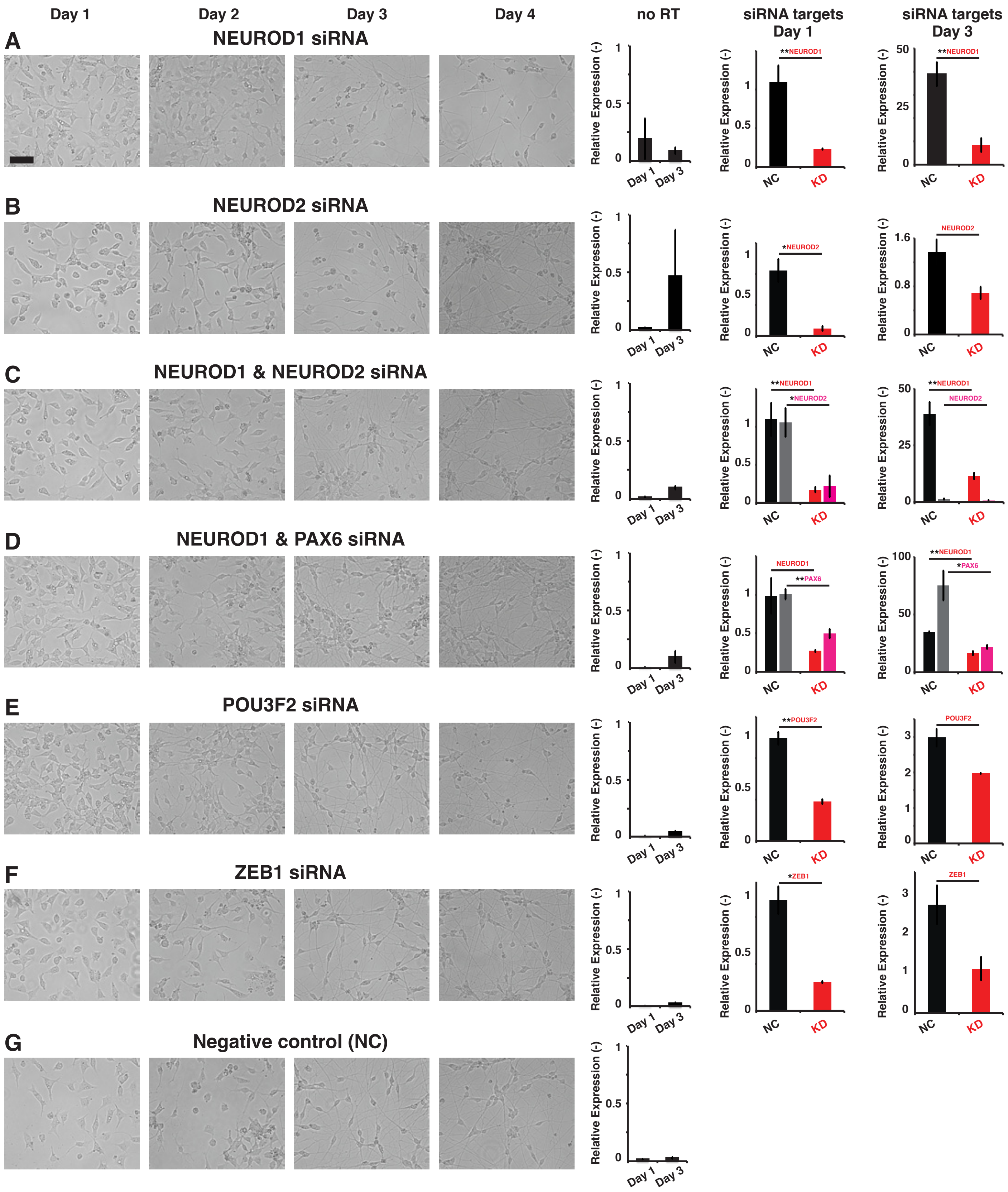

Day 1
Day 2
Day 3
Day 4
no RT
siRNA targets Day 1
siRNA targets Day 3
A
NEUROD1 siRNA
B
NEUROD2 siRNA
C
NEUROD1 & NEUROD2 siRNA
D
NEUROD1 & PAX6 siRNA
E
POU3F2 siRNA
F
ZEB1 siRNA
G
Negative control (NC)
Relative Expression (-)
NC
KD
**NEUROD1
*NEUROD2
NEUROD2
**PAX6
*PAX6
NEUROD1
**POU3F2
POU3F2
*ZEB1
ZEB1

Figure S13 - Comparison of individual Neurogenins and the iNGN cassette. Differences in gene expression resulted when comparing cells derived using by the iNGN construct (Neurog1+Neurog2) and doxycycline-induced constructs with NEUROG1 or NEUROG2. (**A**) NEUROG1 (upper panel) and NEUROG2 (lower panel) both individually differentiated PGP1 iPS cells into MAP2-positive (green) neurons after four days of induction. Scale bar, 100μm. (**B**-**D**) Microarray gene expression analysis of neurons (day 4) generated by the iNGN or individual Neurogenin constructs. (**B**) After correction for multiple hypotheses, 1732 and 305 genes were differentially expressed for the NEUROG1 and NEUROG2 cells, respectively, when compared to the iNGN cells. Significantly differentially expressed genes are plotted in red, and GO terms that were significantly enriched in differentially expressed genes are shown to the right (Fisher's exact test, $p < 0.001$).

Figure S13

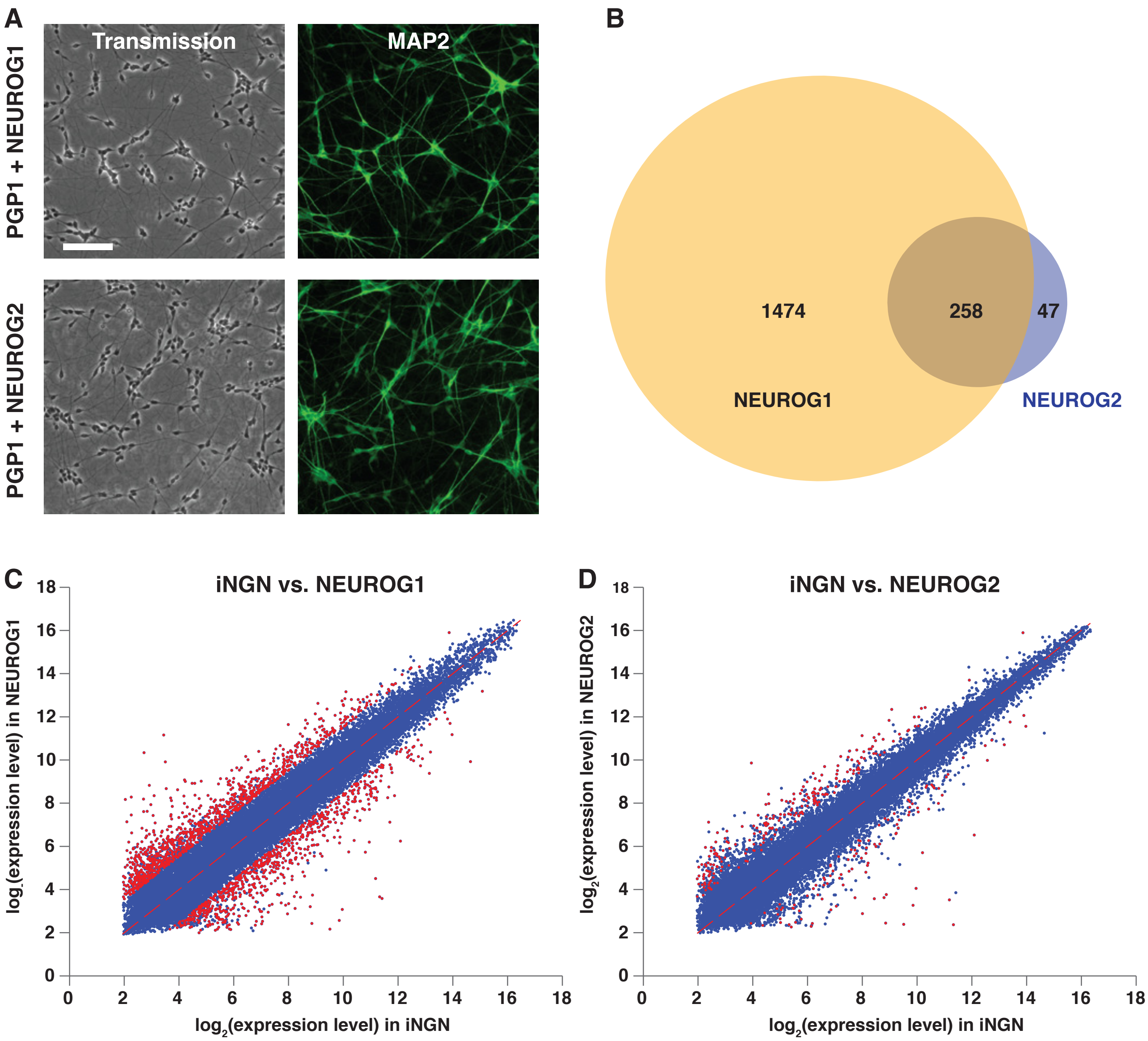
A
Transmission
MAP2
PGP1 + NEUROG1
PGP1 + NEUROG2
B
1474
258
47
NEUROG1
NEUROG2
C
iNGN vs. NEUROG1
log2(expression level) in NEUROG1
log2(expression level) in iNGN
D
iNGN vs. NEUROG2
log2(expression level) in NEUROG2
log2(expression level) in iNGN
